# Visual Identification of Light-Driven Breakage of the Silver-Dithiocarbamate Bond by Single Plasmonic Nanoprobes

**DOI:** 10.1038/srep15427

**Published:** 2015-10-23

**Authors:** Peng Fei Gao, Bin Fang Yuan, Ming Xuan Gao, Rong Sheng Li, Jun Ma, Hong Yan Zou, Yuan Fang Li, Ming Li, Cheng Zhi Huang

**Affiliations:** 1 Key Laboratory of Luminescent and Real-Time Analytical Chemistry (Southwest University), Ministry of Education, College of Pharmaceutical Sciences, Southwest University, Chongqing 400716, China; 2 College of Chemistry and Chemical Engineering, Southwest University, Chongqing 400715, China

## Abstract

Insight into the nature of metal-sulfur bond, a meaningful one in life science, interface chemistry and organometallic chemistry, is interesting but challenging. By utilizing the localized surface plasmon resonance properties of silver nanoparticles, herein we visually identified the photosensitivity of silver-dithiocarbamate (Ag-DTC) bond by using dark field microscopic imaging (iDFM) technique at single nanoparticle level. It was found that the breakage of Ag-DTC bond could be accelerated effectively by light irradiation, followed by a pH-dependent horizontal or vertical degradation of the DTC molecules, in which an indispensable preoxidation process of the silver was at first disclosed. These findings suggest a visualization strategy at single plasmonic nanoparticle level which can be excellently applied to explore new stimulus-triggered reactions, and might also open a new way to understand traditional organic reaction mechanisms.

Thiol always play important roles in the modification of thiophilic noble metal nanomaterials, such as the gold and silver nanoparticles, for preparing surfaces with tunable physicochemical properties or biological recognition sites^1^,^2^,^3^. Due to the interatomic S-S distances ideal for epitaxial adsorption onto thiophilic metals and the confirmed excellent stability of Au-DTC bond, dithiocarbamate (DTC) structures have been widely used to modify the gold surfaces with molecules containing amino group^4^,^5^,^6^,^7^,^8^,^9^. However, essentially characteristics of Ag-DTC bond has rarely been researched although there has been several applications of Ag-DTC^10^,^11^.

When single plasmonic nanoparticles used as probes, Dark field microscopic imaging (iDFM) technique^12^,^13^,^14^, as an important complement scattering analysis technology to the surface plasmon spectroscopy technology^15^,^16^, has several unique advantages owing to the localized surface plasmon resonance (LSPR) scattering features, such as accurate localized information and high continuity and time resolution. In such case, iDFM technique at single nanoparticle level has found exciting applications in various fields, including real-time tracking of bioorganisms^17^, real-time monitoring of alloying^14^, identifying an critical intermediate in galvanic exchange reactions^13^, and monitoring the click reaction^12^.

Herein, we present an unreported light-driven breakage of Ag-DTC bond at the single nanoparticle level with iDFM technique, during which an indispensable preoxidation process is disclosed intuitively and plays important roles. Due to the LSPR features of silver nanoparticles (AgNPs) can be readily modulated by the sulfidation^18^,^19^, iDFM technique can be used as the *in situ* indication signal of the Ag-DTC bond breakage reactions in which sulphide species are generated (Fig. 1a). That is to say, the Ag-DTC bond breakage and the followed cleavage of the monosubstituted DTC can be real time monitored *in situ* and visually. To our knowledge, there has been rarely tries to monitor the breakage process of a chemical bond with iDFM technique, and the results showed that the non-scanning iDFM technique could be expected to achieve some new discovery of previously unknown reactions and the mechanism of some traditional reactions.

## Results and Discussion

### Silver nanoprobe design for visual observation of Ag-DTC bond breakage

**Figure 1:**
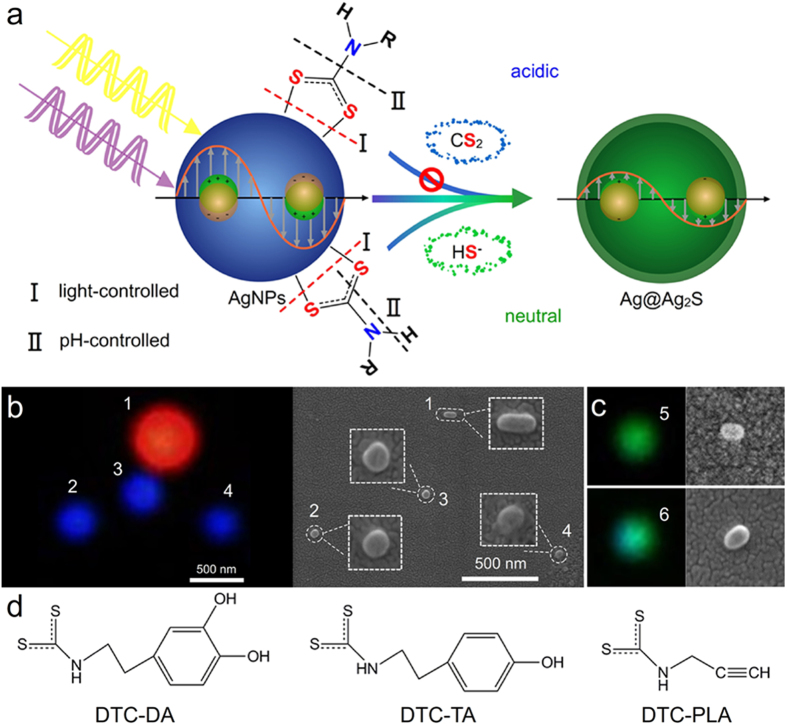
Schematic of light-driven breakage of Ag-DTC bond, the relationship between morphology and scattering light of the nanoprobes and the used DTCs. (**a**) Light induced breakage of the Ag-DTC bond and the following pH-dependent vertical and horizontal cleavage of DTC. (**b**) The co-localization of silver nanospheres and nanorods in DFM image (left) and SEM image (right). (**c**) The co-localization of two nanoparticles scattering green light in DFM image (left) and SEM image (right). (**d**) Structures of three used monosubstituted DTCs, including the dopamine-DTC (DTC-DA), tyramine-DTC (DTC-TA) and propargylamine-DTC (DTC-PLA).

AgNPs, the used single nanoprobes, were at first prepared following the reported protocol^20^ with some adjustments and characterized comprehensively (see Supplementary Experimental section, Supplementary Fig. S1 and Supplementary section for details). The LSPR scattering features of AgNPs in visible range are morphology-dependent^21^. As the probes, spherical AgNPs usually scatter blue light^22^ (so called blue silver nanospheres next) while the rod-shaped AgNPs with the aspect ratio of ~2.5–4 always scatter red light^23^ (so called red silver nanorods next) (Fig. 1b). As the green colour is an intermediate one of the sliver nanospheres during the light-driven reaction, the AgNPs scattered green light (Fig. 1c), which are always ellipsoids and short rods, are not suitable as the nanoprobe for the monitoring of the light-driven reaction.

To investigate the Ag-DTC bond breakage comprehensively, we observed the LSPR scattering features of silver nanoprobes functionalized with three different monosubstituted DTCs (Fig. 1d), including DTC-DA prepared with dopamine (DA) and CS_2_ 24, DTC-TA prepared with tyramine (TA) and CS_2_, and DTC-PLA prepared with propargylamine (PLA) and CS_2_ 25 (see Supplementary Experimental sections for details).

### Light-driven breakage of Ag-DTC bond

**Figure 2:**
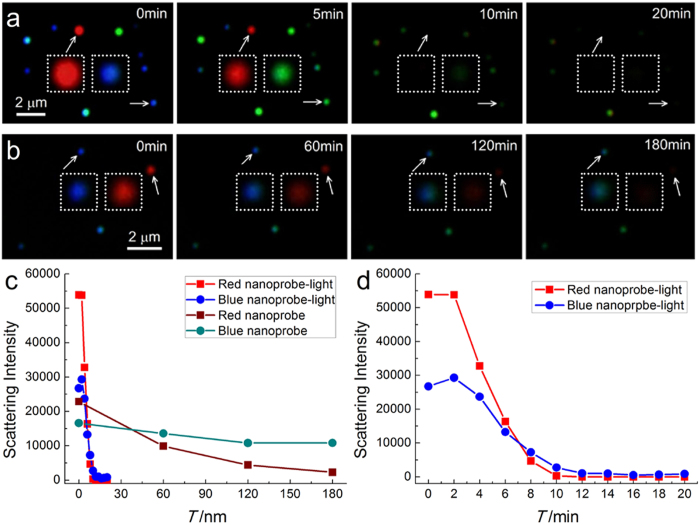
iDFM of the silver nanoprobes during the light induced breakage of Ag-DTC bond. DFM images of (**a**) light-induced and (**b**) natural breakage without light irradiation of Ag-DTC-DA in 0, 5, 10 and 20 min, respectively. The images in square are enlarged one of the nearby nanoparticles pointed with arrows. (**c**) The fast scattering intensity change process of the red nanoprobe (red square), blue nanoprobe (blue circle) in (**a**), and the slow scattering intensity change process red nanoprobe (wine square), blue nanoprobe (cyan circle) in (**b**). (**d**) The scattering intensity change process of probes in (**a**) with an enlarged horizontal axis (within 20 min).

After exposed to the halogen light source of dark field microscope for 20 minutes, blue silver nanospheres turned to green, then to yellow-green, in company with the scattering intensity got increased firstly, and then decreased until too weak to be detected (Methods section, Fig. 2a,c,d, Supplementary Fig. S2 and Movie S1). Differently, the change progress of red silver nanorods was very simple and the scattering intensity simply reduced and the red colour directly disappeared in a fast rate. Although silver nanorods have higher refractive index (RI) response than sliver nanospheres^22^ and a faster response ratio (Fig. 2d), herein nanospheres are more suitable as imaging probes since nanospheres have a wide LSPR redshift and colour change field during the light-driven bond breakage (Supplementary Fig. S3).

To ensure the photosensitivity of Ag-DTC, a control experiment of Ag-DTC-DA without light irradiation was made, which showed a greatly reduced reaction rate (Methods section, Fig. 2b,c), suggesting that the LSPR change was light-dependent, while other that AgNPs without DTCs showed high stability in both the scattering intensity and the colour within 30 minutes (Supplementary Fig. S4a), proving DTC molecule is the prerequisite in the LSPR changes. Similar phenomena were also found in DTC-TA and DTC-PLA (Supplementary Fig. S5, S6). Intriguingly, the LSPR changing rate of Ag-DTC-PLA was obviously accelerated with a follow-up irradiation after the dark condition (Supplementary Fig. S6b,c and e), further identifying the photosensitivity.

### Morphologic and composition analysis of single silver nanoprobe after the reaction

**Figure 3:**
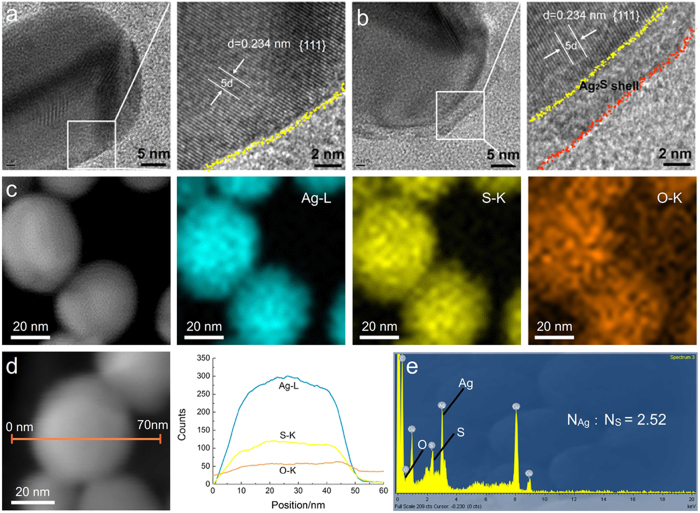
Morphologic imaging and element analysis after the light-driven Ag-DTC breakage in neutral condition. HR-TEM image of (**a**) silver nanoparticle and (**b**) silver nanoparticle after the reaction. (**c**) STEM-HAADF image and Ag, S and O mapping of Ag@Ag_2_S nanospheres. (**d**) STEM-HAADF image of a single Ag@Ag_2_S nanosphere and EDXS line profile. (**e**) EDXS point profile of the Ag_2_S shell.

In order to clarify the details of the reaction and the role that the light played in the acceleration of the reaction rate, comprehensive studies helpful to reveal light-controlled reaction mechanism, including the elemental analysis, influencing factors and the critical preoxidation, were carried out. TEM image of single silver nanoprobe in Fig. 3a showed a typical lattice of silver (111) lattice plane^26^ with a spacing of 2.34 Å, and an obvious ~3 nm shell without lattice was formed on the surface of the nanoprobe after the reaction (Fig. 3b and Supplementary Fig. S7).

Elemental analysis of the core-shell structure showed Ag and S elements were homogeneously distributed (Supplementary Fig. 3c–e), while the O element mapping had a low signal-to-noise ratio, indicating a low oxygen abundance. The lowest Ag/S ratio about 2.52 at edge position of the nanoparticle from the energy-dispersive X-ray spectroscopy (EDXS) was higher than in Ag_2_S, deducing that there should be some unreacted Ag atoms. The HRTEM imaging and elemental analysis confirmed that an Ag@Ag_2_S structure was formed after the light-driven Ag-DTC breakage.

### Degradation of released DTC

In the presence of oxygen, sulfidation of the silver by S^2−^ can be described with equation (1) 27,28, and the sulfidation by HS^−^ and H_2_S be with equation (2 and 3) 29,


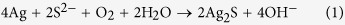



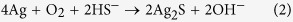



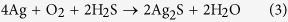


The distribution of sulphide species, including S^2−^, HS^−^, H_2_S, is pH-dependent, and H_2_S is the main form at pH 4.5, while HS^−^ is the main one at pH 7.2^29^, so the sulfidation of Ag at these two pH undergoes pathways as equation (2) and (3), respectively. As the refractive indices of Ag_2_S (~2.2) is much higher than that of Ag (~0.2), the formed Ag_2_S shell on the surface of silver nanoprobe can lead a redshift of LSPR peaks.


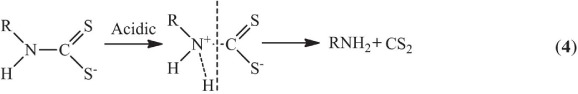



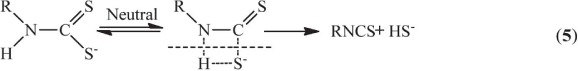


**Figure 4:**
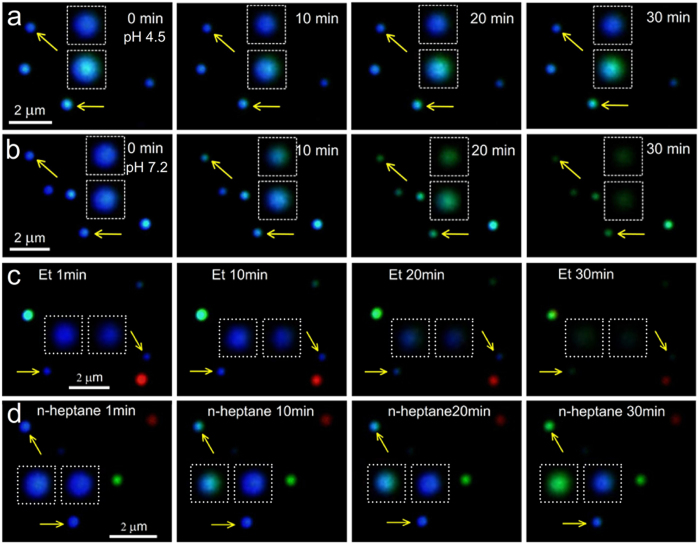
Dark field microscopic images of pH-controlled Ag-DTC breakage in Ag-DTC-DA and solvent-dependent degradation. Dark field microscopic images of Ag-DTC-DA (**a**) in pH 4.5 and (**b**) pH 7.2 Britton-Robinson buffer with light irradiation during 30 min. Dark field microscopic images of Ag-DTC-DA in (**c**) ethanol (Et) and (**d**) n-heptane with light irradiation during 30 min. The images in square from (**a**–**d**) are the corresponding enlarged images of the near particles pointed with arrows.

As equation (4) shows, DTC can undergo a vertical acidic decomposition, resulting in CS_2_ and the amine molecules^30^,^31^. In neutral condition, however, another horizontal cleavage mode is also possible, as equation (5) shows, generating HS^−^ and isothiocyanate (ITC). Usually, the decomposition conditions of DTC into ITCs are often harsh or result in intractable byproducts^32^, so the presence of Ag should be a driving force for the horizontal cleavage of the DTC forming HS^−^. It’s the sulfidation capability of the generated HS^−^ and CS_2_ at different pH that directly leads to the pH-dependent dynamic response of LSPR changes (Fig. 4a,b; see Supplementary Fig. S8 and S9 for the large area images). Generally, the rate of the sulfidation of Ag by H_2_S/HS^-^ is an order of magnitude greater than that by CS_2_ 33, so the LSPR of silver nanoprobes can be changed quickly at pH 7.2. In addition, the reaction rate also shows a positive correlation to the polarity (Fig. 2a and Fig. 4c,d, see Supplementary Fig. S2, Fig. S10 and S11 for the large area images and see Supplementary section for details).

### Critical preoxidation in the sulfidation process

**Figure 5:**
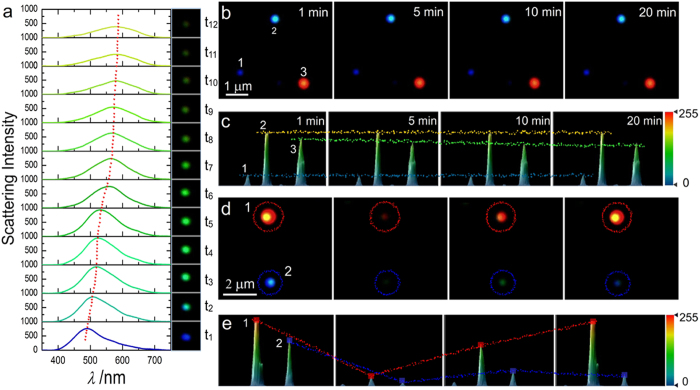
iDFM and spectra of silver nanospheres during the light-driven Ag-DTC breakage and the inhibition of sulfidation process with sodium ascorbate (SA) and the reverse reaction with NaBH_4_. (**a**) Simultaneous iDFM and spectroscopic measurements of a single nanosphere during the light-driven reaction. (**b**) The iDFM image of treatment with 100 mM SA. (**c**) The scattering intensity of the nanoparticles in (**b**). (**d**) From left to right are images before the reaction, after the reaction, treated with 100 mM NaBH_4_ for 15 minutes and further treated with 500 mM NaBH_4_ for 15 minutes. (**e**) The corresponding scattering intensities of the four situations in (**d**).

Single nanoparticle scattering spectra indicated that blue silver nanospheres had a redshift more than 100 nm and the intensity initially increased and further decreased, while red silver nanorods had a narrow redshift in red colour range and a simply decreased intensity (Fig. 5a and Supplementary Fig. S12).

It was found there is an important preoxidation link of Ag contained in the sulfidation process of Ag as shown equation (6) 28,


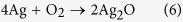


To confirm the preoxidation process, 100 mM sodium ascorbate (SA), a commonly used reducing agent and the pH of which was nearly neutral, was employed to inhibit the preoxidation process (see Supplementary Experimental section for details). As Fig. 5b,c showed, the two silver nanospheres (No. 1 and 2) were stable within 20 minutes, and the silver nanorod (No. 3) showed a 10.8% decrease in scattering intensity, suggesting that the preoxidation process was very important in the suifidation of Ag. The reduction potential of Au was much higher than Ag (E_0_(Au^3+^/Au) = 1.50 V; E_0_(Ag^+^/Ag) = 0.80 V)^34^, making Au-DTC be rather stable^4^,^5^ and non-photosensitive (Supplementary Fig. S13).

To further understand the LSPR change, sodium borohydride (NaBH_4_), a strong reducing agent, was employed to act with the Ag@Ag_2_S. As Fig. 5d,e showed, after treatment with NaBH_4_ in 100 and 500 mM chronologically, the nearly disappeared red and blue dots gradually turned clear and the red value of the red nanoprobe recovered to 89.13%, but the blue value of the blue nanoprobe only recovered to 17.59% (Supplementary Table S1). Intriguingly, that the scattering intensity of the nanosphere turned weak slightly when changed to blue colour from the green colour was nearly an inverse process of the light-induced LSPR change (Fig. 5a), so the NaBH_4_ should lead to an inverse reaction process of the sulfidation and the formed Ag atoms adsorb to the particle surface, showing the blue LSPR feature.

### Mechanism of the light-induced dissociation of Ag-DTC bond

**Figure 6:**
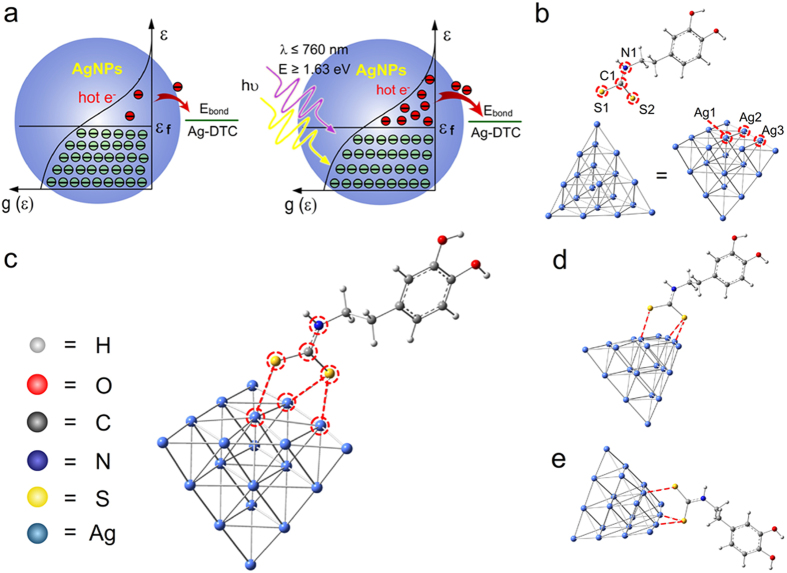
The light induced hot electrons and the calculated binding energy of Ag-DTC by Gaussian simulation. (**a**) Schematic of Fermi-Dirac type distribution (at 300 K) of electrons. (**b**) Optimized existing form of DTC-DA and the used Ag (111) crystal plane. The bond length of the bond between molecules in red circle was compared before and after the bond formation. (**c**) A typical connection form of Ag-DTC on the Ag (111) crystal plane. Other perspectives of this connection form were displayed as (**d**,**e**).

The breakage of Ag-DTC can be driven by both visible and ultraviolet light (365 nm and 254 nm, power is ~10% of visible light) (Fig. 2a, Supplementary Fig. S14, S15). Silver nanoparticles without the DTC modifications under irradiation with 254 and 365 nm for 20 minutes were also investigated to exclude the direct photo-oxidation of silver nanoparticles by the high energy UV lights. The results showed that the silver were stable without any obvious changes judging from the LSPR signals (Supplementary Fig. S16, S17). As known, the electrons on metal surface can be excited by light, and then convert into hot electrons^35^,^36^. According to Pauli Exclusion Principle, the energy distribution of the electrons in metal follows the Fermi-Dirac type distribution as equation (7) 37,


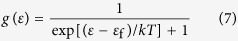


wherein ε_f_ is Fermi level, and k is Boltzmann constant. From Fig. 6a, only few electrons are hot electrons and the number of hot electrons can be added by light irradiation. Hot electrons on metal nanoparticles are always contributed to the strong resonant interaction between surface plasmon and visible photons^35^,^36^. Besides, the ultraviolet irradiation can also excite hot electrons^38^.

As the interband transitions in silver occurs at energies of ~3.8 eV^36^, the hot electrons induced by 254 nm (~4.88 eV) ultraviolet radiation are possibly obtained through the exciting of the interband transitions. The extinction spectrum shows a strong resonant between AgNPs and photons in ~410 nm, but a weak interaction in the ultraviolet region of ~365 nm (Supplementary Fig. S1c). The acceleration by 365 nm irradiation suggests that the electrons with energy over the energy barrier of the reaction may drive the bond breakage.

From the calculation results by the B3LYP DFT method^39^,^40^, the binding energy of DTC on Ag (111) crystal plane showed in Fig. 6c was about 0.77 eV, and was lower than that of Au-DTC which was about 1.5 eV^41^. Some other stable connected types were also found (Supplementary Fig. S18b–d) with bond energy of 0.76, 0.78 and 0.50 eV, respectively. The formation the Ag-DTC could be confirmed with the bond length changes of the C-N and C-S bonds (Supplementary Table S2, S3). So both the visible light (400–760 nm) with energy gap from 1.63 to 3.10 eV and the shorter ultraviolet light can supply enough energy to form hot electrons and drive the Ag-DTC bond breakage.

## Discussion

It’s based on the iDFM technology and single plasmonic nanoprobes that the light-driven breakage of Ag-DTC bond is investigated and several main influence factors, such as light irradiation, pH, solvents polarity and reducing agents, are studied comprehensively. This identification of the photosensitive nature of Ag-DTC may promote the wide application of Ag-DTC bond and the formed Ag@Ag_2_S, a conductor@semiconductor nanocomposites, may be used as a complex thermal conductivity model due to the unique negative correlation between the conductivity and temperature of Ag_2_S.

Up to now, DFM has been rarely used for monitoring of light-driven and multiple regulated reactions. More broadly, this method may open a new way to visually investigate unclear chemical reactions or nature of some chemical bonds. Accordingly, future efforts will be directed to screen other new stimulus-triggered reactions in the field of noble metal, organic sulfur chemistry and organometallic chemistry.

## Methods

### Chemicals

Dopamine hydrochloride, tyramine hydrochloride, propargylamine were obtained from Sigma-Aldrich. Silver nitrate was from Shanghai Shenbo Chemical Co., Ltd. (3-aminopropyl)-trimethoxysilane (APTMS), sodium ascorbate and sodium diethyldithiocarbamate trihydrate were purchased from Aladdin Chemistry Co., Ltd. Carbon disulfide and all the solvents were AR grade and were from Kelong Chemical Reagent Co., Ltd. Commercial slide glass (25.4 × 76.2 mm, 1–1.2 mm in thick) and cover glass (24 × 50 mm, 0.13–0.17 mm in thick) were used for convenience. The copper net covered by a thin pure carbon support film used for the TEM and elements analysis was purchased from Beijing Zhongjingkeyi Technology Co., Ltd.

### Characterization methods

Scanning electron microscopy (SEM) was carried out with a Hitachi S-4800 field emission scanning electron microscope. UV-visible absorption spectra of AgNPs were measured with a Hitachi U-3010 spectrophotometer (Japan). Dark-field microscopic images were captured by a BX51 optical microscope (Olympus, Japan) equipped with a DP72 single chip true-colour chargecoupled device (CCD) camera (Olympus, Japan). The dark field microscope was equipped with a 100 W halogen light source (U-LH100-3). A dark-field condenser (U-DCW, 1.2–1.4) and a 100× object lens were used in the iDFM. The scattering spectra of single nanoparticle were obtained by connecting a spectrograph (MicroSpec-2300i, Roper Scientific) and an intensified CCD camera (PI-MAX, Princeton Instrument) mounted onto the BX51 dark-field optical microscope. The HRTEM imaging was detected with the Tecnai G2 F20 transmission electron microscope (FEI, America, 200 kV) and the equipped energy-dispersive X-ray spectrometer (EDXS) systems and highangle angular-dark-field (HAADF) detector were used for the high-resolution electron microscopy (HREM) imaging and composition analysis. The ultraviolet light of 365 and 254 nm was supplied by the WFH 204B hand-held UV lamp (Shanghai JingKe Industrial Co., Ltd.), and the lamp was removed from the lamp-box and fixed in the place of dark field microscopy light source to achieve the UV irradiation of sample under dark field microscopy.

### Real-time monitoring of the light- and pH-controlled reaction

In order to real-time monitor the light- and pH-controlled reaction, a home-made sample cell with a thickness suitable for loading solvent and focusing was prepared with slide glass and cover glass as our previous reported methods^14^. The changes of the LSPR signal of the AgNPs in different states were recorded by dark field microscopic images and scattering spectra. The effects of light on the reaction were obtained by imaging with the irradiation of the white light source of the microscope or keeping the samples in dark conditions but only turn on the light when it was need to take the images.

The scattering spectra of single nanoparticle was achieved by regulation effect of the silt in the optical path, and the nearby signal of the area was used for the background correction. To better record the fast change process, the amount of the connected DTC-DA was reduced to 1/3. To investigate the effect of pH on the reaction, the solvent was changed to the buffer with the corresponding pH. In the whole imaging, the solvent medium was all controlled to a volume of 150 μm.

### Single nanoparticle imaging data processing

The scattering intensity changes of the red and blue nanoprobe in the light-driven breakage process or in dark condition in Fig. 2 was analyzed by the Image Pro Plus 6.0 software (IPP 6.0). To comparing the scattering intensities in the whole process, the intensities at each time point was firstly counted by the “Image histogram” of IPP 6.0. The colour mode was chosen as “HSI” to count the intensity without the considering of the colour, such as the red, green or blue. The intensity of nanoparticles in Fig. 5 was counted in the same method. The “RGB” mode of IPP 6.0 was used to count the blue or red colour value of the single nanoprobe when treated with the reducing agents.

## Additional Information

**How to cite this article**: Gao, P. F. *et al.* Visual Identification of Light-Driven Breakage of the Silver-Dithiocarbamate Bond by Single Plasmonic Nanoprobes. *Sci. Rep.* **5**, 15427; doi: 10.1038/srep15427 (2015).

## Supplementary Material

Supplementary Information

Supplementary Movie S1
