# Supplementary material for: Visual Identification of Light-Driven Breakage of the Silver-Dithiocarbamate Bond by Single Plasmonic Nanoprobes
Source: Sci Rep. 2015 Oct 23;5:15427. doi: 10.1038/srep15427 (PMC4616019; doi:10.1038/srep15427)
Supplement: Supplementary Information [file srep15427-s1.doc]

**Supplementary Information**

**for**

**Visual identification of light-driven breakage of the silver-dithiocarbamate bond by single plasmonic nanoprobes**

Peng Fei Gao,^1^ Bin Fang Yuan,^2^ Ming Xuan Gao,^2^ Rong Sheng Li,^1^ Jun Ma,^2^ Hong Yan Zou,^1^ Yuan Fang Li,^2^ Ming Li^2^ & Cheng Zhi Huang^1,2*^

^1^Key Laboratory of Luminescent and Real-Time Analytical Chemistry (Southwest University), Ministry of Education, College of Pharmaceutical Sciences, Southwest University. Chongqing 400716, China.

^2^College of Chemistry and Chemical Engineering, Southwest University. Chongqing 400715, China.

^*^Email:  [chengzhi@swu.edu.cn.](mailto: chengzhi@swu.edu.cn. )

**Supplementary Information**

**Table of contents** **Pg**

1. Experimental section. S3-S4

2. Supplementary section. S5

3. Figures for supplementary information. (Figure S1-S18) S6-S15

4. Tables for supplementary information. (Table S1-S3) S16

5. Captions for supplementary movies. (Movie S1) S17

6. References  S17

**Experimental section.**

**Preparation of AgNPs.**

The silver nanoparticles were prepared by the glycerol assisted method^1^ with some modifications. In brief, a 50 mL glycerol/water mixture (20 vol% glycerol) was stirred (1000 rpm) in a 100 mL conical flask and heated up to 95°C. Then to the solvent, 8.5 mg silver nitrate was added followed by 0.8 mL sodium citrate (3%) 1 min later. After 10 min, to the formed AgNPs, 200 μl 50 mM AgNO_3_ and 100 μl sodium citrate (3%) were added added successively, and this operation was repeated twice to achieve a continued growth. Stop heating after the solvent was stirred for another 15 min at 95°C since the last addition and keep stirring until the solvent cooled to room temperature. The prepared AgNPs were stored at 4^o^C. AgNPs of other sizes could be obtained by changing the adding times of AgNO_3_ and sodium citrate which had a positive correlation with the size of the silver nanoparticels.

**Synthesis of DTC-DA and DTC-TA.**

DTC-DA was prepared following the previous reported synthetic schemes with some modifications. Dopamine hydrochloride (0.300 g, 1.58 mmol) was partially dissolved in THF (4 mL) containing Et_3_N (0.240 mL, 1.72 mmol), and then methanol (3.5 mL) was slowly added with stirring. When the solid was solved, CS_2_ (100 μl, 1.58 mmol) was added dropwise under ice cold conditions, and the solution was left stirring for 2 h in the ice water bath. A light yellow colour developed soon after the addition of CS_2_, and the colour changed deep with the increase of reaction. The preparation of DTC-TA was through the same operation.

**Synthesis of DTC-PLA.**

DTC-PLA was synthesized according to the previous report^2^. Briefly, 0.600 g (10.9 mmol) propargylamine (PLA) was dissolved in 30 ml of dry hexane in a 50 ml flask. To this solvent, 11 g (109 mmol) Et_3_N was added. The flask was placed under N_2_ atmosphere and cooled to 0^o^C to buffer the exothermic reaction. Slowly, 1.26 g (16.4 mmol) CS_2_ was added. Then the cooling bath was removed and the reaction was kept stirring at room temperature for 1 h. The white product DTC-PLA was formed and separated with chromatographic column.

**Assemble of the DTC monolayer to the AgNPs.**

As the light- and pH-controlled reaction was underwent on the slide glass, the AgNPs diluted to 1/200 of their original concentration were firstly deposited to the slide glass functionalized with APTMS for 10 min, after that, the excess AgNPs were rinsed away with deionized water. Then the slide glass containing AgNPs were soaked into an ethanol solution of the prepared DTC-DA or DTC-PLA in the concentration of 26.3 mM for 40 min to complete the connection of monosubstituted DTC to the AgNPs.

**Effect of SA on the light-controlled reaction and treatment of Ag@Ag_2_S with NaBH_4_.**

The effect of SA on the light-induced bond breakage reaction was studied in the same operation as the monitoring of the LSPR change process described above with a small adjustment that the reaction medium was changed to 100 mM SA, instead of the water used before.

To further investigate the formed Ag_2_S shell with the treatment of NaBH_4_, the scattering signals of the red nanorod and blue nanosphere were recorded by the iDFM, firstly, and their intensities were defined as 100%. Then the state of the nanoparticles after the light-induced reaction of Ag-DTC-DA was imaged by the dark field microscopy. After that, the glass slide containing nanoparticles was handled with 100 mM NaBH_4_ in ice-cold water for 15 min followed by the imaging of the same two nanoparticles. This progress was repeated once but the concentration of NaBH_4_ was turned to 500 mM.

All the colour value and the scattering intensity of the single nanoparticles were obtained with the image pro plus 6.0 software.

**Computational details.**

All calculations were performed with DFT as implemented in Gaussian 09 package. As we know that the hybrid B3LYP approach was widely used in theoretical calculations since it generally leads to more accurate energies. The geometry optimization was optimized using the mixed basis, the LANL2DZ basis set for Ag and the 6-31G basis set for the remaining atoms. Vibrational frequency calculations done at the B3LYP/6-31G level of theory were used to verify all of the stationary points (NIMAG 0). In addition, the relative Gibbs free energies in vacuum, which are corrected with the vibrational zero-point energies (ZPE), were discussed in present article.

**Supplementary section.**

**Characteristics of the silver nanoprobes.**

The shapes of the prepared AgNPs are mainly spheres with an average size of about 45 nm and a few are rods from the SEM and TEM images in Figure S1a and b. As Figure S1c showed, the extinction spectrum which has a sharp peak in ~408 nm is also in accordance with the sphere shape and concentrated distribution of particle sizes. The co-localized observation of the same single silver nanoparticles by combining dark-field microscopy with scanning electron microscopy confirmed the relationship between the shapes and scattering colours. The results in Figure 1c showed that AgNPs scattering red and blue light were rods (particle 1) and spheres (particle 2-4), respectively. The green dots under the dark field microscopy were usually ellipsoid or short rod which had a LSPR between the spheres and rods (Figure 1d). The single nanoparticle scattering spectra also revealed the green coloured nanoparticles had a wavelength between that of the blue and the red nanoparticles.

**Effect of the solvent polarity on the light-driven reaction rate.**

As equation (**1**-**3**) shows, the sulfidation of silver is in relation to consumption or generation of water or OH^-^, so the sulfidation rate of AgNPs is obviously regulated by the proton level of the medium. A series of solvents with different polarity including water, ethanol and n-heptane were chosen as reaction medium. As Figure 4c and d showed, the sulfidation rate slowed down with the reduction of polarity, comparing to that in water medium (Figure 2a). The enlarged images in Figure 4d showed that only partial of silver nanospheres underwent the sulfidation reaction, indicating a slow reaction rate. The slow rate under weak polar condition could also be seen by the comparison of the iDFM of the red particles in these three solvents.

**Figures for supplementary information.**


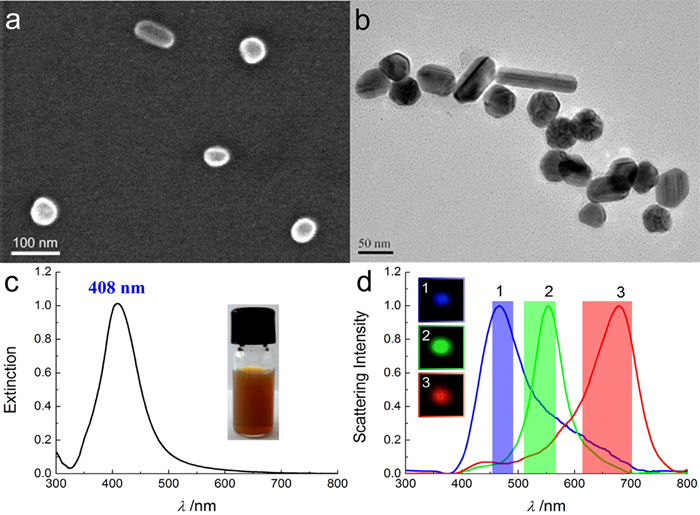


**Figure S1.** Characteristics of silver nanoparticles. (a) SEM image and (b) TEM image of the silver nanoparticles (c) Extinction spectrum and the photograph of silver nanoparticles. (d) The dark field microscopic image of nanoparticles scattering light of three-primary colours and their corresponding single nanoparticles scattering spectra.


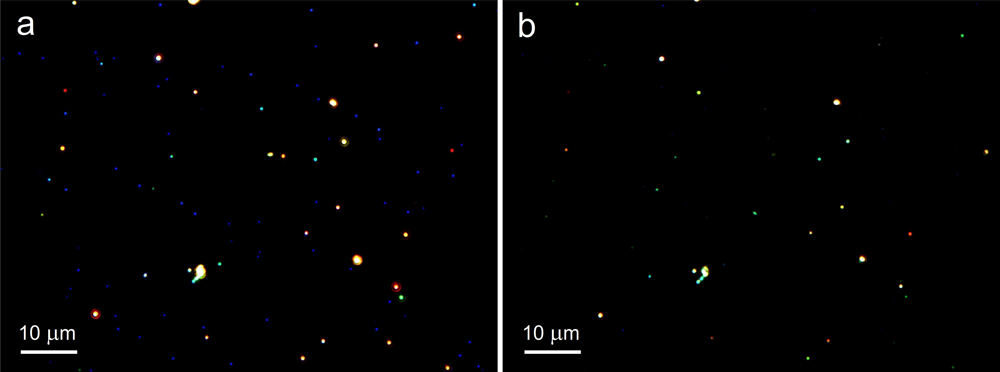


**Figure S2.** Dark field microscopic images of Ag-DTC-DA of the same area before (a) and after (b) irradiation with dark filed white light for 20 min. Most of the silver nanoparticles, both the nanospheres and nanorods, almost disappeared after the irradiation.


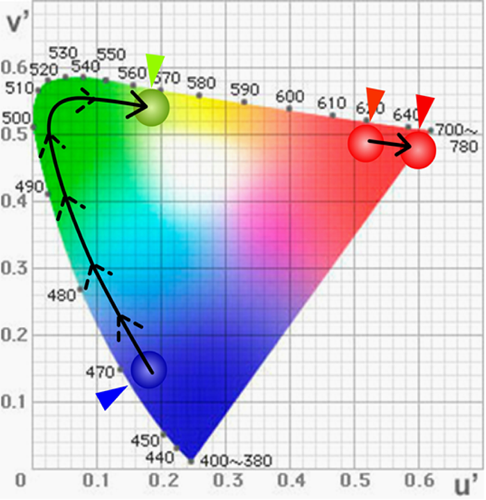


**Figure S3** Schematic of the colour change range of blue nanosphere and red nanorod on the CIE u’−v’ chromaticity diagram. The nanosphere underwent a more significant colour change than the nanorod, so it could display more detailed process of reaction.


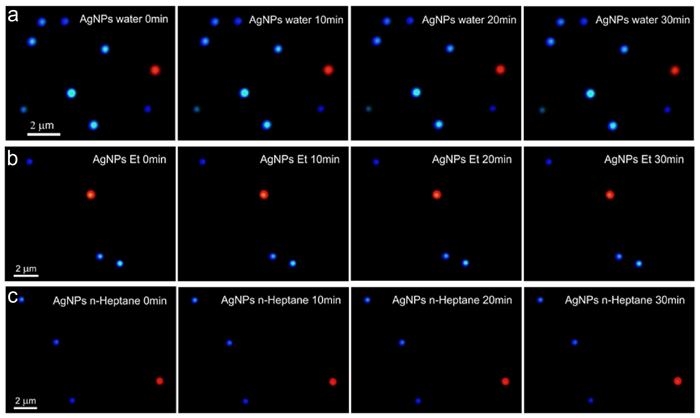


**Figure S4.** Dark field microscopic images of the silver nanoparticles without functionalization with the DTC in different media for 30 min. The reaction and observation media were (a) water, (b) ethanol and (c) n-heptane, respectively. During 30 min, there were no obvious changes of the LSPR of the silver nanospheres and nanorods compared with the silver nanoparticles connected with the DTC molecules.


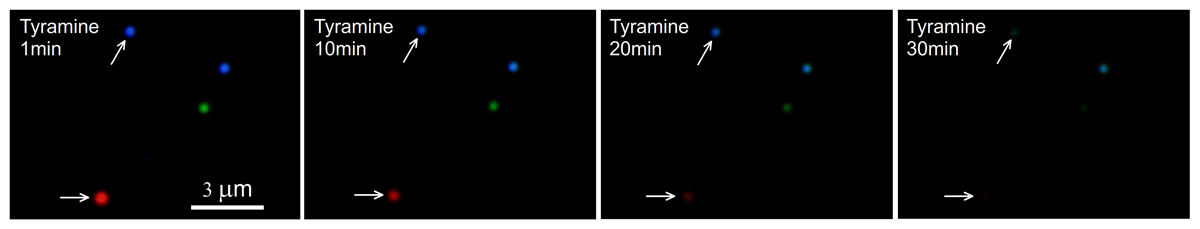


**Figure S5.** Dark field microscopic images of Ag-DTC-TA after irradiation with the dark field white light for 30 min. Both the LSPR of nanospheres and the nanorods turned weak and nearly disappeared in 30 min, and the change rate of the LSPR is slower than the LSPR change induced by the DTC-DA.


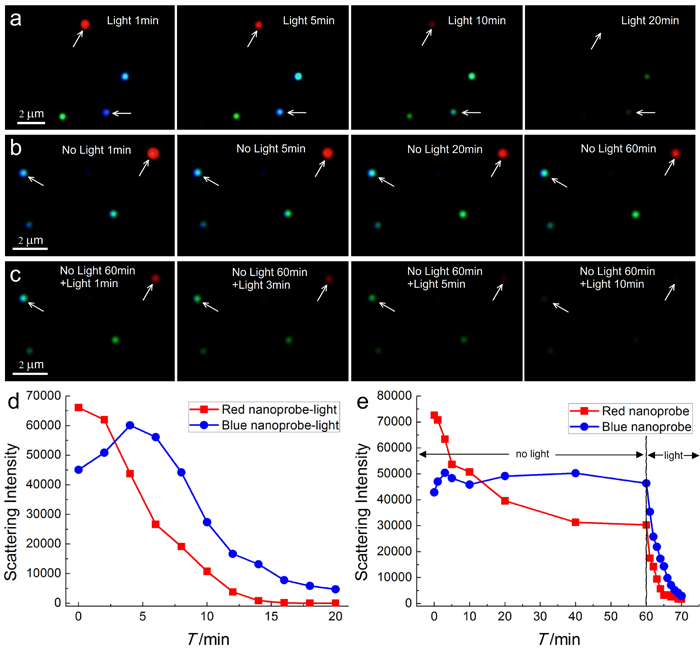


**Figure S6.** Dark field microscopic images of the Ag-DTC-PLA (a) with or (b) without the irradiation with the halogen light. With the irradiation of 20 min, the silver nanoparticles disappeared completely while the silver nanoparticles without irradiation were still red or kept blue ingredient in 60 min. (c) When the particles were changed to under light irradiation for 10 min after the dark condition of 60 min in (b), the LSPR of the nanospheres and nanorods disappeared quickly. (d) Scattering intensity change curves of the single red and blue nanoprobe in (a). (e) Scattering intensity change curves of the single red and blue nanoprobe under dark condition in 60 min in (b) and was changed under light irradiation for 10 min in (c).


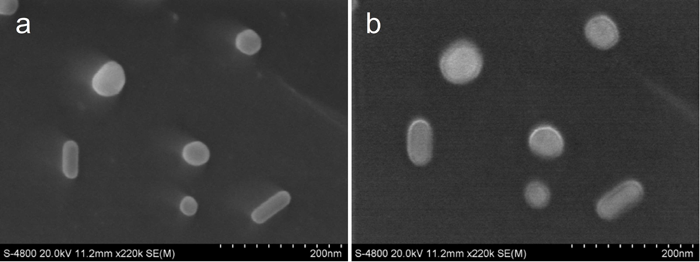


**Figure S7.** SEM of the silver nanoparticle before and after the light-controlled reaction. The silver nanoparticles with the treatment of DTC-DA (a) before and after (b) the irradiation of 365 nm ultraviolet lamp with a power of 12 w for 30 min in water and the whole reaction process is on the monocrystalline silicon substrate used for SEM. There was no obvious difference in the morphology but the conductivity of the particle surface turned down which may be due to the generation of amorphous Ag_2_S shell with a low conductivity on the surface.


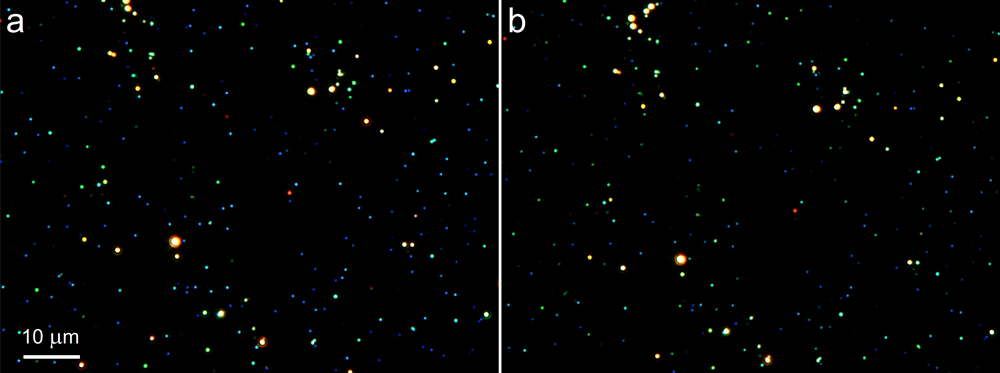


**Figure S8.** Dark field microscopic images of Ag-DTC-DA of the same area in pH 4.5 before (a) and after (b) irradiation with dark filed white light for 30 min.


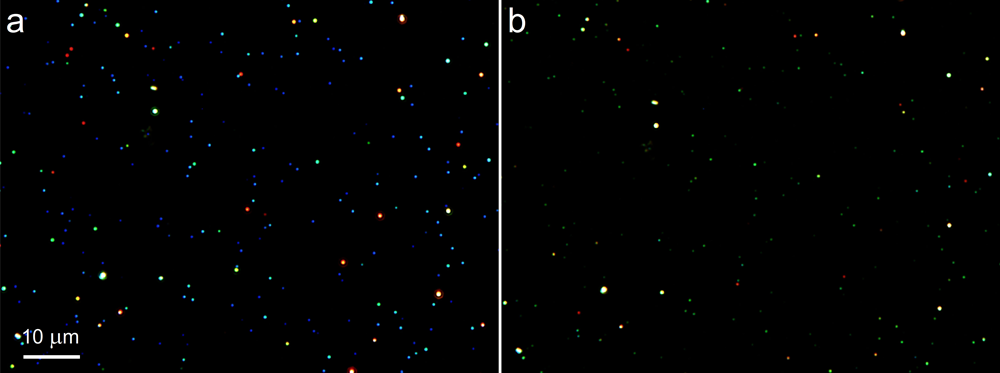


**Figure S9.** Dark field microscopic images of Ag-DTC-DA of the same area in pH 7.2 before (a) and after (b) irradiation with dark filed white light for 30 min. The LSPR signal in the neutral pH changes much quickly than in pH 4.5.


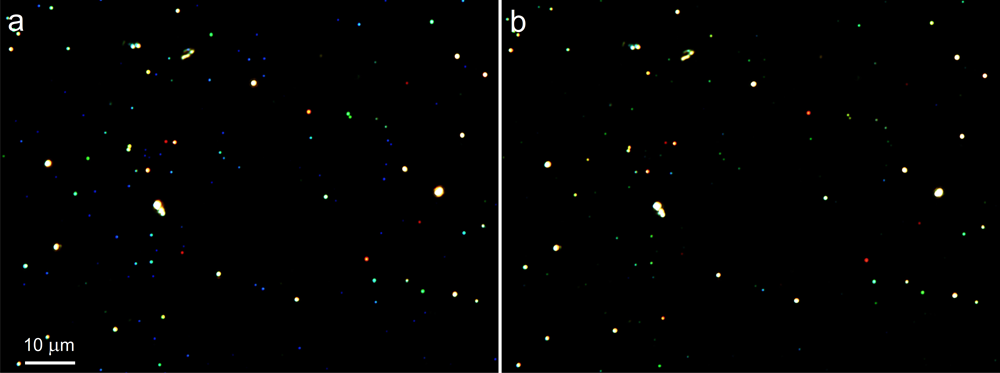


**Figure S10.** Dark field microscopic images of Ag-DTC-DA of the same area before (a) and after (b) irradiation with dark filed white light for 30 min in ethanol.


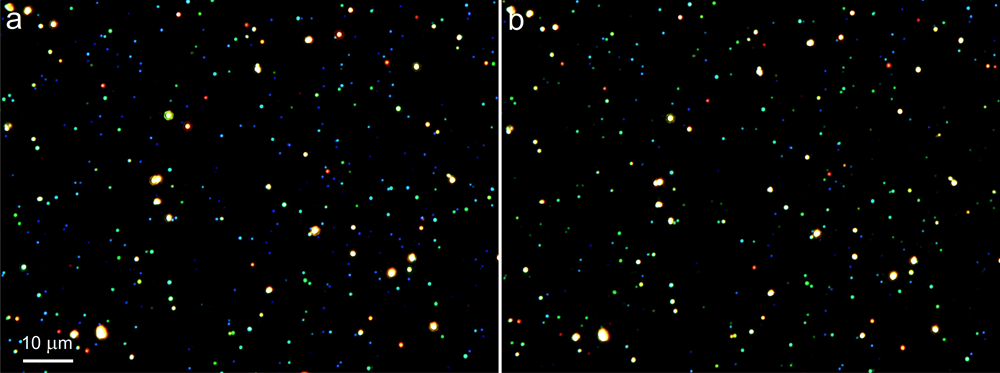


**Figure S11.** Dark field microscopic images of Ag-DTC-DA of the same area before (a) and after (b) irradiation with dark filed white light for 30 min in n-heptane. The LSPR change rate turned lower with the decrease of the solvent polarity.


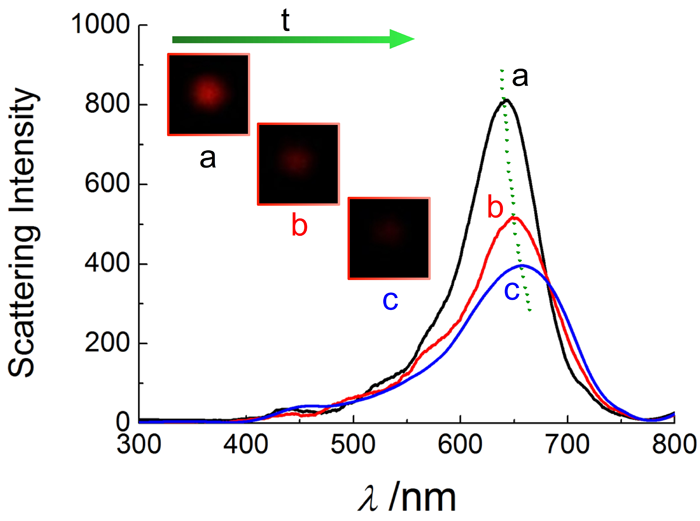


**Figure S12.** Simultaneous dark-field scattering microscopic images and spectroscopic determination of a single nanorod during the light-controlled reaction process. Different from the spectroscopic change process of the blue nanosphere, there was only a simple decrease of the intensity and a redshift of the scattering peak.


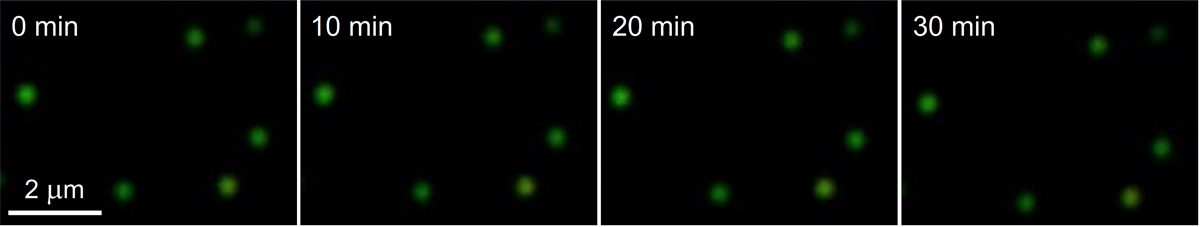


**Figure S13.** Dark field microscopic images of Au-DTC-DA after irradiation with the dark field white light for 30 min. No obvious changes can be seen from the iDFM results during the 30 min, suggesting that the Au-DTC bond should be more stable than the Ag-DTC and the Au-DTC is not photosensitive.


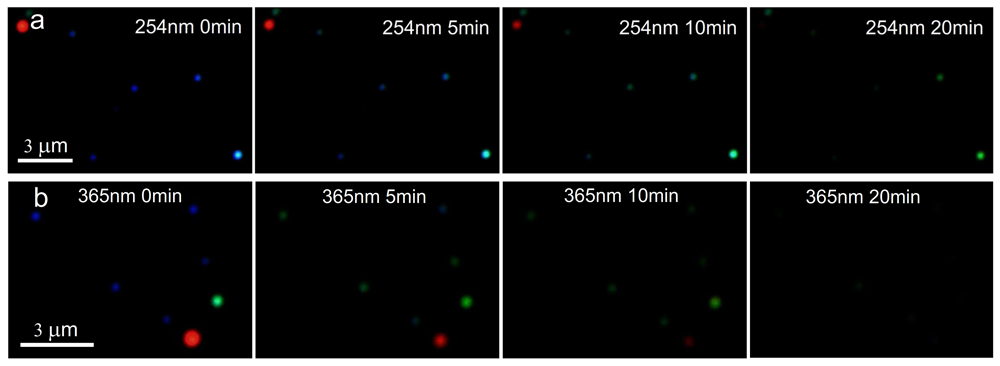


**Figure S14.** Dark field microscopic images of the silver nanoparticles with the irradiation of (a) 254 nm and (b) 365 nm UV-light with a power of 11 w under the dark field condenser for 20 min. Although the power of the UV-light is much slow than the dark field white light, both the UV-light in 254 nm and 365 nm could lead to a fast LSPR change rate which might be due to the UV-light with a higher energy than the visible light according to the equation **E=hc/ λ**.


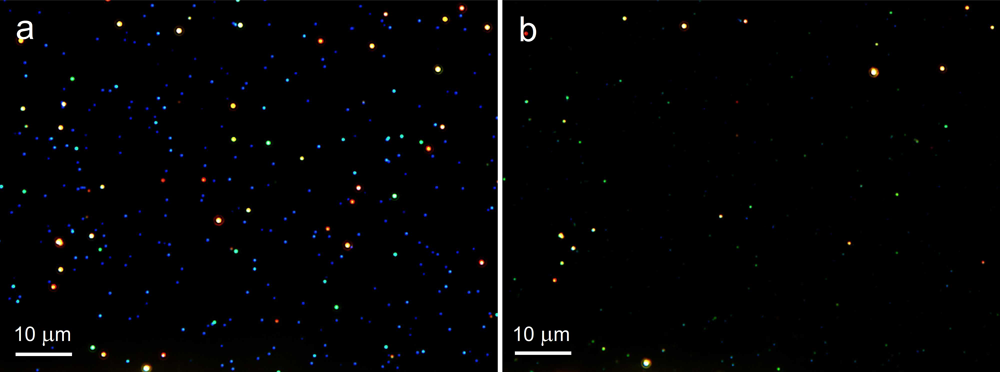


**Figure S15.** Dark field microscopic images of Ag-DTC-DA of the same area before (a) and after (b) irradiation with 365 nm UV-light of 11 w under the dark field condenser for 30 min. All the silver nanoparticles turned weak and some had been disappeared. The TEM and element analysis sample was prepared in this condition which led to the sulfidation of nanoparticles without obvious morphology changes.


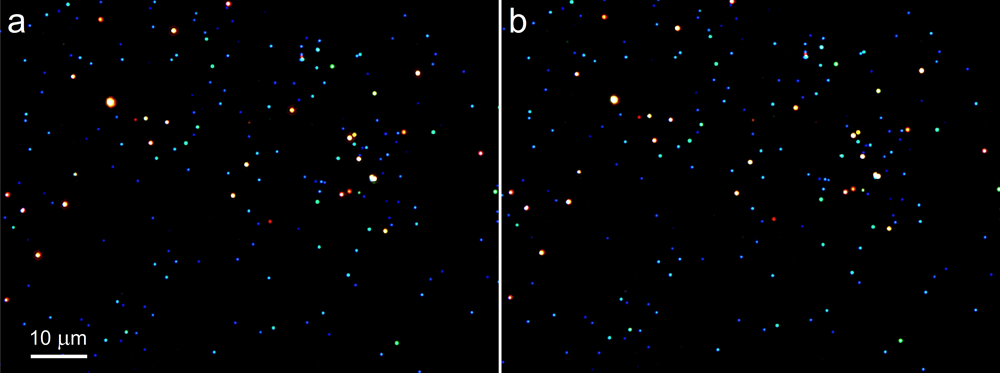


**Figure S16.** Dark field microscopic images of AgNPs of the same area before (a) and after (b) irradiation with 254 nm UV-light of 11 w under the dark field condenser for 30 min. The LSPR signal of the silver nanoparticles without modification are stable under the UV-light irradiation.


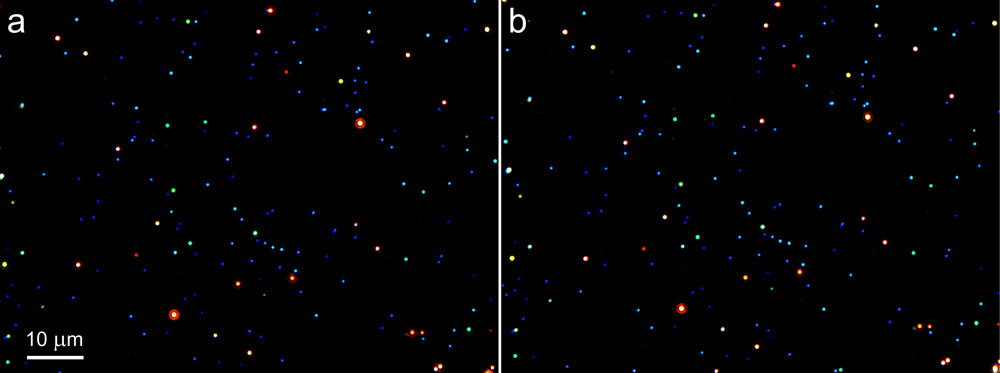


**Figure S17.** Dark field microscopic images of AgNPs of the same area before (a) and after (b) irradiation with 365 nm UV-light of 11 w under the dark field condenser for 30 min. The LSPR signal of the silver nanoparticles without modification are also stable under this UV-light irradiation.


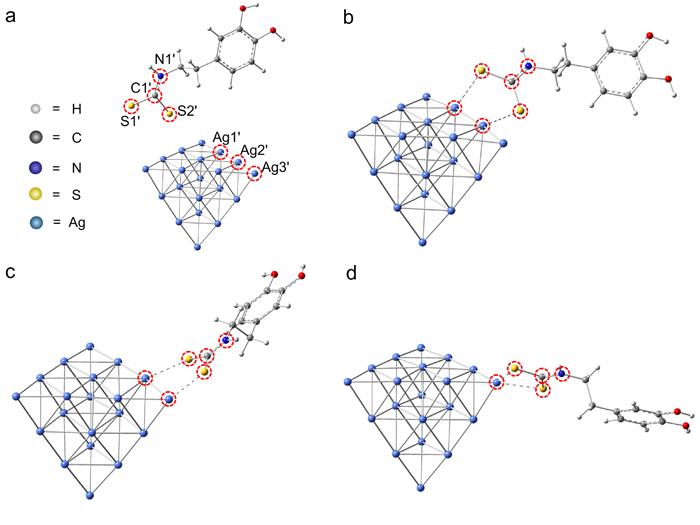


**Figure S18.** Three other stable connection types of DTC on Ag (111). (a) To better display the connection differences of these three types, the related molecules were numbered. The two sulfur atoms was connected to (b) two silver atoms on the edge, (c) two silver atoms on the edge and the vertice and (d) the same silver atom on the vertice.

**Tables for supplementary information.**

**Table S1.** Blue colour value of the blue nanosphere and red nanorod in different statements. Four states in chronological order, including before the light-induced reaction, after the light-induced reaction, treated with 100 mM NaBH_4_ and then treated with 500 mM NaBH_4_, of these two kinds of probe are analyzed. Both of these two probes showed a recovery of the colour value after the treatment with NaBH_4_.

| Probe state | Before reaction | After reaction | 100mM  NaBH_4_ | 500 mM  NaBH_4_ |
| --- | --- | --- | --- | --- |
| Red value of nanorod /% | 100.0 | 5.358 | 39.38 | 89.13 |
| Blue value nanosphere /% | 100.0 | 1.003 | 3.120 | 17.59 |

**Table S2.** Bond length of the bonds between the numbered molecules in Figure 6 (b) before and after the formation of the Ag-DTC bond. The bond length of all these bonds turned longer after the formation the Ag-DTC bond.

| Bond | C1-N1 | C1-S1 | C1-S2 | Ag1-Ag2 | Ag1-Ag3 | Ag2-Ag3 |
| --- | --- | --- | --- | --- | --- | --- |
| Bond length (before, Å) | 1.336 | 1.758 | 1.753 | 2.917 | 2.916 | 2.813 |
| Bond length (after, Å) | 1.343 | 1.770 | 1.804 | 3.023 | 3.072 | 3.074 |

**Table S3.** Bond length of the C-N and C-S bonds after the formation of the Ag-DTC bond in the three types in Figure S11. The bond length of all these bonds also turned longer than before the formation the Ag-DTC bond.

| Bond | C1’-N1’ | C1’-S1’ | C1’-S2’ |
| --- | --- | --- | --- |
| Bond length (Figure 11 (b) , Å) | 1.346 | 1.791 | 1.772 |
| Bond length (Figure 11 (c) , Å) | 1.347 | 1.779 | 1.789 |
| Bond length (Figure 11 (d) , Å) | 1.345 | 1.789 | 1.784 |

**Captions for supplementary movies.**

**Movie S1.** Video microscopy of a field of several DTC-DA connected single Ag nanoprobes, including both red silver nanoprobe and blue silver nanoprobe, undergoing a light-driven Ag-DTC breakage. The light was the white halogen light source of the dark field microscopy. In a period of 20 min, the LSPR scattering of the silver nanoprobes underwent an intensity decrease and red shift of the peak as Figure 2 showed.

References

1. Schlücker, S. & Steinigeweg, D. Monodispersity and Size Control in the Synthesis of 20-100 nm Quasi-Spherical Silver Nanoparticles by Citrate and Ascorbic Acid Reduction in Glycerol/Water Mixtures. *Chem. Commun.* **48,** 8682-8684 (2012).

2. Heugebaert, T. S. A., Vervaecke, L. P. D. & Stevens, C. V. Gold(iii) chloride catalysed synthesis of 5-alkylidene-dihydrothiazoles. *Org. Biomol. Chem.* **9,** 4791-4794 (2011).
